# Influence of elevated liver enzyme level on 30-day mortality rates in patients undergoing nonemergency orthopedic surgery

**DOI:** 10.1186/s13741-024-00395-7

**Published:** 2024-05-06

**Authors:** Tzu-Ruei Liao, Yuan-Wen Lee, Chuen-Chau Chang, Alan Hsi-Wen Liao, Yen-Chun Lai, Chih-Chung Liu

**Affiliations:** 1 Department of Anesthesiology, Taipei Medical University Hospital, No. 252, Wuxing St, Taipei, 11031 Taiwan; 2 Department of Anesthesiology, School of Medicine, College of Medicine, Taipei Medical University, Taipei, Taiwan; 3 Anesthesiology and Health Policy Research Center, Taipei Medical University Hospital, Taipei, 110 Taiwan

**Keywords:** Liver enzymes, Aminotransferase, Orthopedic surgery, Surgical outcomes

## Abstract

**Background:**

The effect of elevated preoperative liver enzyme levels on postoperative outcomes is a topic of concern to clinicians. This study explored the association between elevated preoperative liver enzyme levels and surgical outcomes in patients undergoing orthopedic surgery.

**Methods:**

Using the American College of Surgeons National Surgical Quality Improvement Program database, we obtained data on adult patients who received nonemergency orthopedic surgery under general anesthesia between 2011 and 2021.

**Results:**

We evaluated the data of 477,524 patients, of whom 6.1% (24 197 patients) had elevated preoperative serum glutamic oxaloacetic transaminase (SGOT) levels. An elevated SGOT level was significantly associated with 30-day postoperative mortality (adjusted hazard ratio, 1.62; 95% confidence interval, 1.39 to 1.90). We determined that the mortality rate rose with SGOT levels. The results remained unchanged after propensity score matching.

**Conclusion:**

Elevated preoperative SGOT levels constitute an independent risk factor for 30-day postoperative mortality and are proportionately associated with the risk of 30-day postoperative mortality.

**Supplementary Information:**

The online version contains supplementary material available at 10.1186/s13741-024-00395-7.

## Background

The effects of anesthesia and surgery on patients with mildly elevated liver enzyme levels are obscure (Sahin et al. 2007). Clinicians may be concerned about the perioperative risks of patients with unexpectedly elevated liver enzymes before surgery. However, further evaluation of abnormal liver function tests may result in delayed surgery and increased medical costs. Patients with elevated preoperative liver enzyme levels generally present as asymptomatic. The identification of patients who require further evaluation is challenging. Accordingly, the influence of elevated preoperative liver enzyme levels on postoperative outcomes warrants investigation.

A systematic review reported a 10% to 21.7% prevalence rate of elevated liver enzyme levels in the general population. Similar results have been reported by studies on a Taiwanese cohort (Radcke et al. 2015; Chen et al. 2007). Elevated liver enzyme levels are not uncommon in surgical patients. In two German cohort studies, the prevalence of elevated preoperative aminotransferase levels ranged from 10 to 20% in patients undergoing orthopedic surgery (Wiegand et al. 2006; Lobstein et al. 2008). Matheson et al. noted elevated serum glutamic-oxaloacetic transaminase (SGOT) and serum glutamic pyruvic transaminase (SGPT) levels in patients undergoing elective hip or knee arthroplasty at 1 week postoperatively; the levels returned to their normal range at 6 weeks postoperatively (Matheson et al. 2009; Quinlan et al. 2012). However, evidence regarding the relationship between elevated postoperative aminotransferase levels and postoperative outcomes is limited.

Researchers have documented the association between elevated aminotransferase levels and increased risk of mortality in the general population. Individuals with elevated aminotransferase levels have been reported to have a 1.2 to 3 times higher risk of all-cause mortality compared with those with normal aminotransferase levels (Arndt et al. 1998; Lee et al. 2008). Even in individuals with normal liver enzyme levels, aminotransferase levels between 20 and 39 IU/L were associated with a 3.3- to 18.2-fold greater risk of mortality due to liver disease compared with aminotransferase levels below 20 IU/L (Kim et al. 2004). However, postoperative outcomes in patients with unexpectedly elevated liver enzyme levels who undergo nonemergency orthopedic surgery remain poorly understood. Accordingly, further investigation is warranted to determine the risk stratification in orthopedic patients with elevated preoperative liver enzyme levels.

The aim of the present study was to explore the association between preoperative elevated liver enzyme levels and 30-day postoperative mortality in patients who received orthopedic surgery. This exploration was conducted using American College of Surgeons National Surgical Quality Improvement Program (ACS-NSQIP) data.

## Methods

### Data source

The ACS-NSQIP is an international program with over 10 participating countries (Ellis and Ko 2017). It contains millions of cases and hundreds of accurate and rigorously collected variables, and it is reviewed periodically. The present study analyzed data from the ACS-NSQIP Participant Use Data Files (ACS-NSQIP PUFs) for the period from 2011 to 2021. Our study was evaluated and approved by the Institutional Review Board of Taipei Medical University (TMU-JIRB No. N202203047).

### Patient selection and characteristics

Adult patients (> 18 years) who received orthopedic surgery between 2011 and 2021 were included in the study. The types of orthopedic surgery included limb amputation, spinal fusion, open reduction of fracture, hip prosthesis, knee prosthesis, and laminectomy. The current procedural terminology (CPT) code for the orthopedic surgeries included is provided in the supplemental content (Supplementary Table 1). Patients who received non-elective surgery and those with missing data on baseline characteristics were excluded. Clinical characteristics including age, sex, functional status, obesity, smoking status, comorbidities (i.e., steroid use, ascites, ventilator dependent, disseminated cancer, diabetes mellitus, hypertension, congestive heart failure, chronic obstructive pulmonary disease (COPD), dialysis, sepsis), and operative information (i.e., type of surgery and American Society of Anesthesiologists (ASA) physical status classification) were obtained from the ACS-NSQIP database.

SGOT and SGPT are both concentrated in the liver. Although SGPT is associated with liver injury, SGPT levels were not included in the ACS-NSQIP database. SGOT is found in the liver, cardiac muscle, skeletal muscle, kidneys, brain, pancreas, lungs, leukocytes, and erythrocytes (Pratt and Kaplan 2000). Although SGOT is less liver specific than SGPT, abnormal SGOT levels may indicate problems involving a wider variety of organ systems and are thus more likely to be associated with postoperative mortality. Consequently, we selected the SGOT as our indicator of postoperative mortality. The upper limit of normal (ULN) for SGOT was defined as 40 IU/L in accordance with previous studies (Liaqat et al. 2021; Aliabadi et al. 2021). Patients were separated into two groups according to the ULN for SGOT level.

Our study outcome was postoperative 30-day all-cause mortality. We further stratified the patients into three groups according to their SGOT level (40 < SGOT ≤ 80, 80 < SGOT ≤ 200, and SGOT > 200) (Kwo et al. 2017). We then analyzed the postoperative 30-day all-cause mortality rates in these subgroups.

### Statistical analysis

Patient baseline characteristics are presented as counts and percentages. The standardized mean difference (SMD) was used to weigh the differences in baseline characteristics between patients with normal liver enzyme level (group 1) and those with elevated liver enzyme level (group 2). An SMD of < 0.1 was considered to indicate a nonsignificant difference between the two groups. We used Cox regression models to assess the hazard ratios (HRs) for postoperative mortality. The HRs were adjusted for all patient characteristics.

Propensity score matching was used to balance the patient characteristics between the two groups (Lunceford 2017). We defined a propensity score as the probability that a patient had an elevated liver enzyme level given observed covariates. A logistic regression model with baseline characteristics was used to estimate propensity scores. We employed propensity score matching as a sensitivity analysis to verify the robustness of our findings.

Statistical significance was indicated at *P* < 0.05. All statistical analyses were performed using the SAS System for Windows 9.4 (SAS Institute, Cary, NC, USA).

## Results

**Fig. 1:**
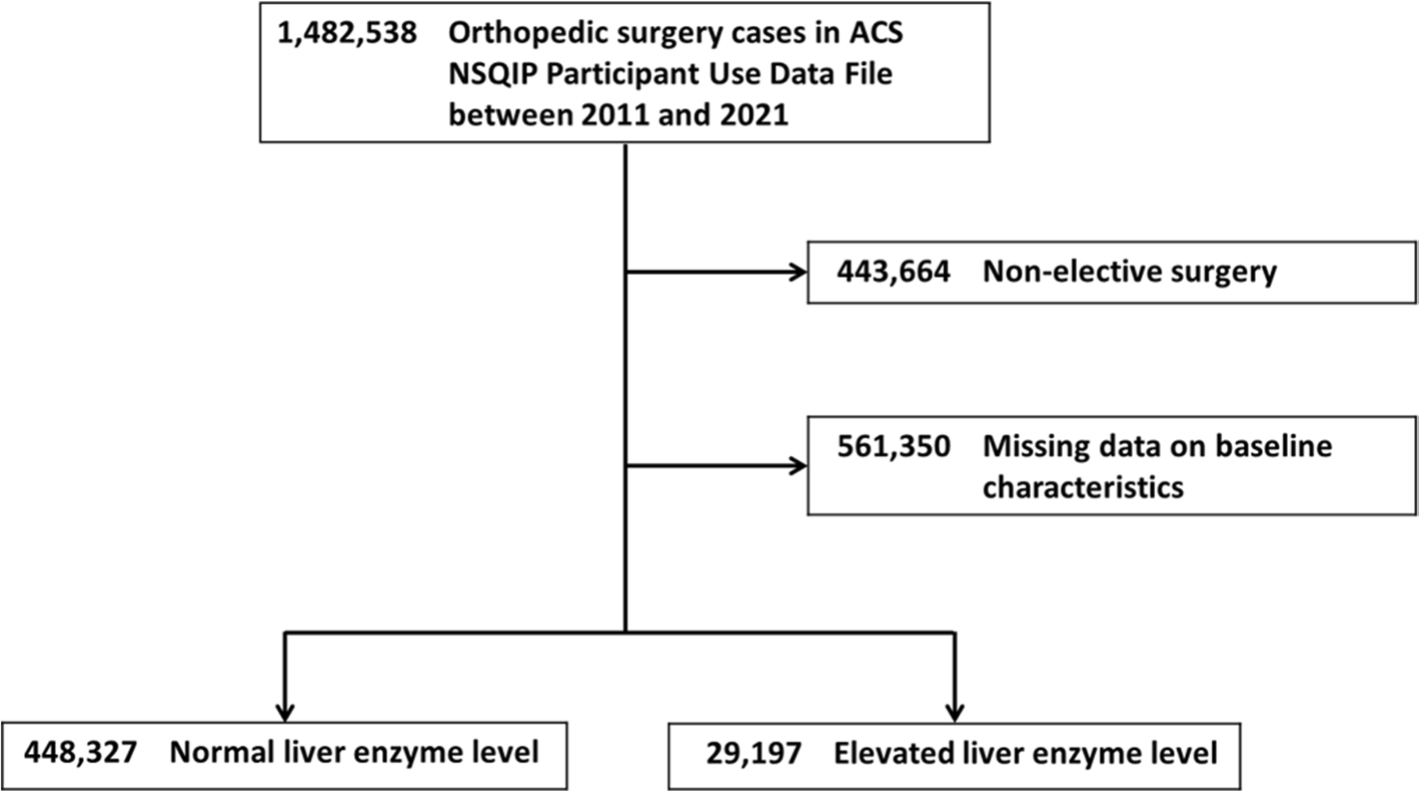
Study sample selection

We identified 1,482,538 patients who received orthopedic surgery between 2011 and 2021. We excluded patients who received nonelective surgery (*n* = 443 664) and those with missing data on baseline characteristics (*n* = 561 350). Ultimately, 477,524 patients who underwent nonemergency orthopedic surgery were included in our analysis (Fig. 1).

Of the patients, 448,327 (93.9%) had normal liver enzyme levels (group 1), and 29,197 (6.1%) had elevated liver enzyme levels. Patient characteristics were similar between the two groups, except for age, sex, smoking status, sepsis, type of surgery, and the ASA classification. The majority of patients in both groups were independent (97.7% in group 1 vs. 96.9% in group 2; SMD: 0.048), had received knee prosthesis (51.0% in group 1 vs. 42.9% in group 2; SMD: 0.163), or had hypertension (61.8% in group 1 vs. 62.6% in group 2; SMD: 0.017). Most of the patients were in ASA classification 2 (47.2% in group 1 vs. 40.4% in group 2; SMD: 0.136) and 3 (48.3% in group 1 vs. 53.8% in group 2; SMD: 0.110). Significant differences in age, sex, smoking status, sepsis, type of surgery, and the ASA classification were observed between the two groups. A greater proportion of patients in group 2 were aged < 65 years (58.8%) than were in group 1 (44.4%; SMD: 0.291). However, group 2 had a lower proportion of patients aged between 65 and 74 years (27.7%) than did group 1 (34.3%; SMD: 0.144), in addition to having a lower proportion of patients aged between 75 and 84 years (10.8%) than did group 1 (17.7%; SMD: 0.198). The two groups did not significantly differ in terms of the proportion of patients aged older than 85 years (3.6% in group 1 vs. 2.7% in group 2; SMD: 0.050). A greater proportion of patients in group 1 were female (41.7%) than did those in group 2 (52.4%; SMD: 0.215); a lower proportion of patients had a smoking habit (11.6% vs. 17.6%; SMD: 0.170); a lower proportion had sepsis (0.7% vs. 1.8%; SMD: 0.101); a lower proportion of patients had open reduction of fracture (5.0% vs. 12.0%; SMD: 0.255); and a greater proportion of patients had received knee prosthesis (51.0% vs. 42.9%; SMD: 0.163; Table 1).

**Table 1:** Characteristics of patients receiving elective orthopedic surgery

	Normal liver enzyme level (*N* = 448,327)		Elevated liver enzyme level (*N* = 29,197)		
Characteristics	*N* (%)		*N* (%)		SMD
Demographics					
Age, years					
< 65	199,061	(44.4)	17,168	(58.8)	0.291
65–74	153,895	(34.3)	8079	(27.7)	0.144
75–84	79,165	(17.7)	3149	(10.8)	0.198
≥ 85	16,206	(3.6)	801	(2.7)	0.050
Sex					
Female	261,338	(58.3)	13,907	(47.6)	0.215
Male	186,989	(41.7)	15,290	(52.4)	0.215
Functional status					
Independent	438,018	(97.7)	28,301	(96.9)	0.048
Dependent	10,309	(2.3)	896	(3.1)	0.048
Obesity	246,832	(55.1)	16,485	(56.5)	0.028
Smoking	52,164	(11.6)	5143	(17.6)	0.170
Comorbidities					
Steroid use	20,427	(4.6)	1394	(4.8)	0.010
Ascites	94	(0.0)	60	(0.2)	0.055
Ventilator dependent	57	(0.0)	32	(0.1)	0.039
Disseminated cancer	2250	(0.5)	390	(1.3)	0.087
Diabetes mellitus	77,525	(17.3)	6174	(21.2)	0.098
Hypertension	276,933	(61.8)	18,279	(62.6)	0.017
Congestive heart failure	2180	(0.5)	221	(0.8)	0.034
COPD	19,466	(4.3)	1511	(5.2)	0.039
Dialysis	1652	(0.4)	123	(0.4)	0.008
Sepsis	3089	(0.7)	529	(1.8)	0.101
Operative information					
Type of surgery					
Limb amputation	1949	(0.4)	237	(0.8)	0.048
Spinal fusion	30,873	(6.9)	2301	(7.9)	0.038
Open reduction of fracture	22,238	(5.0)	3504	(12.0)	0.255
Hip prosthesis	140,844	(31.4)	8810	(30.2)	0.027
Knee prosthesis	228,504	(51.0)	12,510	(42.9)	0.163
Laminectomy	23,919	(5.3)	1835	(6.3)	0.041
ASA classification					
1	9872	(2.2)	542	(1.9)	0.025
2	211,380	(47.2)	11,801	(40.4)	0.136
3	216,359	(48.3)	15,699	(53.8)	0.110
4 or 5	10,716	(2.4)	1155	(4.0)	0.089

*COPD* chronic obstructive pulmonary disease

The number of deaths at 30 days after surgery was 1214 (0.3%) in group 1 and 194 (0.7%) in group 2. Group 2 had a higher mortality rate (adjusted HR, 1.62; 95% CI, 1.39 to 1.90). Group 2 was further divided into three subgroups according to SGOT levels (40 < SGOT ≤ 80, 80 < SGOT ≤ 200, and SGOT > 200). We identified a dose-dependent association between SGOT levels and the HR. The adjusted HRs for mortality in patients with an SGOT level between 40 and 80 IU/L (adjusted HR, 1.41; 95% CI, 1.18 to 1.69), in those with an SGOT level between 80 and 200 IU/L (adjusted HR, 2.09; 95% CI, 1.53 to 2.86), and in those with an SGOT level greater than 200 IU/L (adjusted HR, 6.36; 95% CI, 3.84 to 10.53) increased with the SGOT levels (Table 2).

**Table 2:** Association between liver dysfunction and the risk of mortality in patients receiving elective orthopedic surgery

Liver function	Total number	Number of mortality (%)	Unadjusted HR (95% CI)	Adjusted HR (95% CI)
Elevated liver enzyme level				
No (SGOT ≤ 40)	448,327	1214 (0.3)	1.00	1.00
Yes (SGOT > 40)	29,197	194 (0.7)	2.46 (2.11–2.86)	1.62 (1.39–1.90)
40 < SGOT ≤ 80	24,821	136 (0.6)	2.03 (1.70–2.42)	1.41 (1.18–1.69)
80 < SGOT ≤ 200	3961	42 (1.1)	3.93 (2.89–5.34)	2.09 (1.53–2.86)
SGOT > 200	415	16 (3.9)	14.51 (8.86–23.76)	6.36 (3.84–10.53)

*P* for trend: < 0.0001

Propensity score matching was used to adjust for patient characteristics in the study population. No significant differences in patient characteristics were detected between the two groups after propensity score matching (Table 3). An increased risk of mortality and a dose-dependent association between SGOT levels and the HR were still observed in group 2 after propensity score matching (adjusted HR, 1.60; 95% CI, 1.27 to 2.03; Table 4).

**Table 3:** Characteristics of patients receiving elective orthopedic surgery after 1:1 propensity score matching

	After propensity score matching				
	Normal liver enzyme level (*N* = 29,189)		Elevated liver enzyme level (*N* = 29,189)		
Characteristics	*N* (%)		*N* (%)		SMD
Demographics					
Age, years					
< 65	17,224	(59.0)	17,162	(58.8)	0.004
65–74	8075	(27.7)	8078	(27.7)	< 0.001
75–84	3126	(10.7)	3149	(10.8)	0.002
≥ 85	764	(2.6)	800	(2.7)	0.007
Sex					
Female	13,893	(47.6)	13,904	(47.6)	0.001
Male	15,296	(52.4)	15,285	(52.4)	0.001
Functional status					
Independent	28,243	(97.1)	28,296	(96.9)	0.010
Dependent	846	(2.9)	893	(3.1)	0.010
Obesity	16,519	(56.6)	16,483	(56.5)	0.002
Smoking	5202	(17.8)	5139	(17.6)	0.006
Comorbidities					
Steroid use	1403	(4.8)	1393	(4.8)	0.002
Ascites	40	(0.1)	55	(0.2)	0.015
Ventilator dependent	16	(0.1)	29	(0.1)	0.018
Disseminated cancer	373	(1.3)	389	(1.3)	0.006
Diabetes mellitus	6201	(21.2)	6172	(21.1)	0.003
Hypertension	18,275	(62.6)	18,278	(62.6)	< 0.001
Congestive heart failure	182	(0.6)	219	(0.8)	0.016
COPD	1514	(5.2)	1509	(5.2)	0.001
Dialysis	111	(0.4)	122	(0.4)	0.006
Sepsis	491	(1.7)	525	(1.8)	0.011
Operative information					
Type of surgery					
Limb amputation	216	(0.7)	236	(0.8)	0.009
Spinal fusion	2325	(8.0)	2301	(7.9)	0.003
Open reduction of fracture	3529	(12.1)	3498	(12.0)	0.004
Hip prosthesis	8966	(30.0)	8810	(30.2)	0.003
Knee prosthesis	12,521	(42.9)	12,510	(42.9)	0.001
Laminectomy	1832	(6.3)	1834	(6.3)	< 0.001
ASA classification	10,254	(42.1)			
1	535	(1.8)	542	(1.9)	0.002
2	11,817	(40.5)	11,801	(40.4)	0.001
3	15,759	(54.0)	15,697	(53.8)	0.004
4 or 5	1078	(3.7)	1149	(3.9)	0.014

*COPD* chronic obstructive pulmonary disease

**Table 4:** Association between liver dysfunction and the risk of mortality after 1:1 propensity score matching

Liver function	Total number	Number of mortality (%)	Unadjusted HR (95% CI)	Adjusted HR (95% CI)
Elevated liver enzyme level				
No (SGOT ≤ 40)	29,189	109 (0.4)	1.00	1.00
Yes (SGOT > 40)	29,189	191 (0.7)	1.76 (1.39–2.22)	1.60 (1.27–2.03)
40 < SGOT ≤ 80	24,814	134 (0.5)	1.45 (1.12–1.86)	1.39 (1.08–1.80)
80 < SGOT ≤ 200	3961	42 (1.1)	2.85 (1.99–4.06)	2.05 (1.42–2.95)
SGOT > 200	414	15 (3.6)	9.88 (5.76–16.95)	6.14 (3.54–10.67)

*P* for trend: < 0.0001

## Discussion

This study analyzed the data of 477,524 patients from the ACS-NSQIP database and revealed that an elevated preoperative liver enzyme level is associated with increased postoperative 30-day mortality in patients undergoing nonemergency orthopedic surgery. Moreover, we identified a linear relationship between preoperative SGOT levels and postoperative 30-day mortality. Our data suggest that an elevated preoperative SGOT level is an independent risk factor for 30-day mortality in patients undergoing orthopedic surgery.

According to our data, 6.1% (29 197) of the patients undergoing orthopedic surgery had elevated preoperative SGOT levels. Similarly, Lobstein et al. reported elevated SGOT levels in 7% of 960 patients without evidence of viral hepatitis who underwent orthopedic surgery (Lobstein et al. 2008). Wiegand et al. investigated two hospital orthopedic surgery cohorts and observed elevated SGOT levels in 11.7% of 1454 patients without hepatitis C (Wiegand et al. 2006). Our findings agree with previous reports that elevated preoperative SGOT levels are common in patients undergoing nonemergency orthopedic surgery. Clinicians should not disregard the negative effect of elevated preoperative SGOT levels on postoperative outcomes.

Studies have examined the association between preoperative SGOT levels and surgical outcomes in cardiac surgery, emergency general surgery, and head and neck surgery (Shang et al. 2021; Narueponjirakul et al. 2020; Abt et al. 2018). In adult patients without liver disease who underwent cardiac surgery, a preoperative abnormal SGOT level was an independent risk factor for in-hospital and 90-day mortality (Shang et al. 2021). In octogenarian patients who received emergency general surgery, an elevated preoperative SGOT level increased the risk of in-hospital and 1-year mortality (Narueponjirakul et al. 2020). Moreover, in head and neck surgery, an abnormal preoperative SGOT level increased the risk of surgical-site infection and 30-day postoperative complications but not mortality (Abt et al. 2018). Using the ACS-NSQIP database, Bishop et al. analyzed the data of 815 077 patients with ASA classifications 1 to 3 who received elective surgery between 1992 and 2001 (Bishop et al. 2008). They found that an elevated SGOT level (SGOT > 40 IU/L) was an independent risk factor for 24-h and 30-day postoperative mortality. In contrast to previous studies, our study not only identified SGOT level as an independent risk factor for 30-day postoperative mortality in patients undergoing nonemergency orthopedic surgery but also demonstrated that SGOT levels are proportionately associated with the risk for 30-day postoperative mortality.

Common hepatic etiologies of mildly elevated liver enzyme levels are viral hepatitis, alcoholic liver disease, cirrhosis, steatohepatitis, and medications (toxins). Acetaminophen, nonsteroidal anti-inflammatory drugs, carbamazepine, phenytoin, and trazodone are common analgesics and adjuvants that can cause liver transaminase elevation in patients undergoing orthopedic surgery (Oh et al. 2017). Certain supplements, including shark cartilage and vitamin A, can also result in reversible cases of elevated liver enzyme levels (Giboney 2005). Additional causes of mild liver enzyme elevation include celiac disease, hemolysis, myopathy, hyperthyroidism, strenuous exercise, and macro-aspartate aminotransferase (AST) (Kwo et al. 2017). Clinicians should carefully evaluate surgical patients with elevated liver enzyme levels to identify the possible etiology and correct reversible factors.

According to the American College of Gastroenterology’s clinical guidelines for the evaluation of abnormal liver chemistries, patients with mildly elevated liver enzyme levels (2 to 5 × ULN) should first be assessed for medicine-related causes, fatty liver disease, and viral hepatitis. Recommended evaluations include an iron panel; tests for liver function, infection, and hepatitis; and an abdominal ultrasound. In the case of negative results, clinicians should repeat the tests after 3 months and consider a liver biopsy to test for autoimmune-related causes (Kwo et al. 2017). However, surgical patients with mildly elevated liver enzyme levels are often identified only 1 day prior to surgery, and the completion of extensive evaluations is difficult without delaying the originally scheduled surgery. We recommend that clinicians consider other risk factors. A previous retrospective cohort study indicated that patients with disseminated cancer, poor functional status, a high ASA classification (ASA 3 vs. 1), weight loss > 10%, or ascites had a high odds ratio for 30-day postoperative mortality (Bishop et al. 2008). Further preoperative evaluation should be considered for patients with elevated preoperative SGOT levels and the aforementioned risk factors. In addition, anesthesia type may influence postsurgical outcomes. Although a retrospective study of 91 patients did not detect significant changes between preoperative and postoperative liver enzyme levels (Sahin et al. 2007), a retrospective Korean study on the effects of total intravenous anesthesia (TIVA) and inhalation anesthesia on liver enzyme levels had divergent findings (Oh et al. 2020). Specifically, the Korean study included 730 patients with elevated preoperative liver enzyme levels who underwent surgery with propofol-based TIVA or inhalation anesthesia. The results indicated lower postoperative SGOT and SGPT levels in both anesthesia groups, but they revealed a significantly lower change in SGPT levels after TIVA than that observed after inhalation anesthesia. However, postoperative liver chemistry changes do not share a definite correlation with postoperative outcomes. Clinicians should consider the effect of anesthesia type in patients with elevated preoperative liver enzyme levels.

This study has several limitations. First, we could not observe long-term outcomes because the ACS-NSQIP database contains only 30-day postoperative outcomes. Furthermore, according to ACS-NSQIP data variable definition, preoperative lab values are drawn within 90 days prior to the primary procedure. In our study population, the mean interval between the acquisition of SGOT level and the surgery was 19.75 days. This gap between obtaining the SGOT levels and the surgery date may pose challenges in accurately reflecting the patient’s current condition on the day of surgery. Despite the data gap, our results demonstrated a significant association between elevated SGOT levels and postoperative 30-day mortality among patients undergoing elective orthopedic surgery. Clinicians are therefore advised to allocate time for thorough patient evaluation and to seek expert consultation to address the underlying causes of elevated SGOT levels. Second, we excluded nearly 40% of the patients undergoing orthopedic surgery owing to missing data on preoperative SGOT levels. A previous study recommended that researchers examine the pattern of data gaps and evaluate the pros and cons of different methods for addressing missing data (Parsons et al. 2011). This study only analyzed the population with complete data. However, patients without data on preoperative SGOT levels may not receive liver function tests because of their relatively healthy condition. This may be a source of potential selection bias in our study. Third, the impact of increased preoperative liver enzyme levels on patients undergoing different types of anesthesia remains unexplored. Theoretically, patients under regional anesthesia have less medication exposure than those receiving general anesthesia. This difference may influence the effect of elevated liver enzyme levels on patient outcomes. Fourth, this study exclusively focused on elective orthopedic surgery. The generalizability of our findings to other procedures should be approached with caution. According to the surgical risk classification of 2022 ESC Guidelines, more than 95% of the patients in our study underwent low (minor orthopedic surgery) to intermediate (major orthopedic surgery: hip or spine surgery) risk surgeries (Halvorsen et al. 2022). Despite this, our study yielded significant results even within the cohort of patients undergoing low to moderate-risk surgeries. We anticipate that focusing on high-risk surgeries would further underscore the significance of our findings. Fifth, there exist several differences in patient characteristics between the two groups, such as ASA classification, type of surgery, age, and others. Despite our efforts to adjust all patient characteristics for the primary outcome and conduct propensity score matching, it is possible that bias or confounding factors, which were not detected or adequately adjusted for, may still be present. Finally, the ACS-NSQIP database does not include SGPT levels, which constitute a more specific marker of hepatocellular injury than do SGOT levels. On the contrary, SGOT serves as a broader systemic marker, and the elevation of SGOT levels does not necessarily signify liver pathology. Moreover, SGOT and SGPT levels can help determine the etiologies of abnormal liver function tests. Further research is warranted to investigate the implications of other liver enzyme abnormalities in postoperative outcomes.

## Conclusion

This study identified the 30-day postoperative mortality risk following nonemergency orthopedic surgery in patients with elevated preoperative SGOT levels. Patients with elevated preoperative SGOT levels who received nonemergency orthopedic surgery had a higher risk of 30-day postoperative mortality. A higher preoperative SGOT level was associated with a greater HR for 30-day postoperative mortality. Clinicians should consider additional liver function tests to identify the etiology of abnormal test results in patients.

### Supplementary Information

**Supplementary Material 1. **

## Data Availability

The datasets analysed during the current study are available from the joint first author Yuan-Wen Lee on reasonable request.

## Abbreviations

SGOT: Serum glutamic oxaloacetic transaminase
SGPT: Serum glutamic pyruvic transaminase
ACS-NSQIP: American College of Surgeons National Surgical Quality Improvement Program
PUFs: Participant use data files
CPT: Current procedural terminology
COPD: Chronic obstructive pulmonary disease
ASA: American Society of Anesthesiologists
ULN: Upper limit of normal
SMD: Standardized mean difference
HRs: Hazard ratios
AST: Aspartate aminotransferase
TIVA: Total intravenous anesthesia
