# Supplementary material for: Influence of elevated liver enzyme level on 30-day mortality rates in patients undergoing nonemergency orthopedic surgery
Source: Perioper Med (Lond). 2024 May 6;13:35. doi: 10.1186/s13741-024-00395-7 (PMC11071270; doi:10.1186/s13741-024-00395-7)
Supplement: Supplementary file 1 — Supplementary Material 1. [file 13741_2024_395_MOESM1_ESM.docx]

**Supplementary table 1** CPT codes of the included procedures

| **CPT Codes** | **Procedure Code Descriptions** |
| --- | --- |
| 23900 | Interthoracoscapular amputation (forequarter) |
| 23920 | Disarticulation of shoulder |
| 24900 | Amputation, arm through humerus; with primary closure |
| 24920 | Amputation, arm through humerus; open, circular (guillotine) |
| 24930 | Amputation, arm through humerus; re-amputation |
| 24931 | Amputation, arm through humerus; with implant |
| 25900 | Amputation, forearm, through radius and ulna |
| 25905 | Amputation, forearm, through radius and ulna; open, circular (guillotine) |
| 25909 | Amputation, forearm, through radius and ulna; re-amputation |
| 25920 | Disarticulation through wrist |
| 25922 | Disarticulation through wrist; secondary closure or scar revision |
| 25924 | Disarticulation through wrist; re-amputation |
| 25927 | Transmetacarpal amputation; |
| 25929 | Transmetacarpal amputation; secondary closure or scar revision |
| 25931 | Transmetacarpal amputation; re-amputation |
| 26235 | Partial excision (craterization, saucerization, or diaphysectomy) bone (eg, osteomyelitis); proximal or middle phalanx of finger |
| 26236 | Partial excision (craterization, saucerization, or diaphysectomy) bone (eg, osteomyelitis); distal phalanx of finger |
| 26551 | Transfer, toe-to-hand with microvascular anastomosis; great toe wrap-around with bone graft |
| 26910 | Amputation, metacarpal, with finger or thumb (ray amputation), single, with or without interosseous transfer |
| 26951 | Amputation, finger or thumb, primary or secondary, any joint or phalanx, single, including neurectomies; with direct closure |
| 26952 | Amputation, finger or thumb, primary or secondary, any joint or phalanx, single, including neurectomies; with local advancement flaps (V-Y, hood) |
| 27290 | Interpelviabdominal amputation (hindquarter amputation) |
| 27295 | Disarticulation of hip |
| 27590 | Amputation, thigh, through femur, any level; |
| 27591 | Amputation, thigh, through femur, any level; immediate fitting technique including first cast |
| 27592 | Amputation, thigh, through femur, any level; open, circular (guillotine) |
| 27598 | Disarticulation at knee |
| 27880 | Amputation, leg, through tibia and fibula; |
| 27881 | Amputation, leg, through tibia and fibula; with immediate fitting technique including application of first cast |
| 27882 | Amputation, leg, through tibia and fibula; open, circular (guillotine) |
| 27884 | Amputation, leg, through tibia and fibula; secondary closure or scar revision |
| 27886 | Amputation, leg, through tibia and fibula; re-amputation |
| 27888 | Amputation, ankle, through malleoli of tibia and fibula (e.g., Syme, Pirogoff type procedures), with plastic closure and resection of nerves |
| 27889 | Ankle disarticulation |
| 28124 | Partial excision (craterization, saucerization, sequestrectomy, or diaphysectomy) bone (eg, osteomyelitis or bossing); phalanx of toe |
| 28126 | Resection, partial or complete, phalangeal base, each toe |
| 28160 | Hemiphalangectomy or interphalangeal joint excision, toe, proximal end of phalanx, each |
| 28800 | Amputation, foot; midtarsal (e.g., Chopart type procedure) |
| 28805 | Amputation, foot; transmetatarsal |
| 28810 | Amputation, metatarsal, with toe, single |
| 28820 | Amputation, toe; metatarsophalangeal joint |
| 28825 | Amputation, toe; interphalangeal joint |
| 22532 | Arthrodesis, lateral extracavitary technique, including minimal discectomy to prepare interspace (other than for decompression); thoracic |
| 22533 | Arthrodesis, lateral extracavitary technique, including minimal discectomy to prepare interspace (other than for decompression); lumbar |
| 22548 | Arthrodesis, anterior transoral or extraoral technique, clivus-C1-C2 (atlas-axis), with or without excision of odontoid process |
| 22551 | Arthrodesis, anterior interbody, including disc space preparation, discectomy, osteophytectomy and decompression of spinal cord and/or nerve roots; cervical below C2 |
| 22554 | Arthrodesis, anterior interbody technique, including minimal discectomy to prepare interspace (other than for decompression); cervical below C2 |
| 22556 | Arthrodesis, anterior interbody technique, including minimal discectomy to prepare interspace (other than for decompression); thoracic |
| 22558 | Arthrodesis, anterior interbody technique, including minimal discectomy to prepare interspace (other than for decompression); lumbar |
| 22586 | Arthrodesis, pre-sacral interbody technique, including disc space preparation, discectomy, with posterior instrumentation, with image guidance, includes bone graft when performed, L5-S1 interspace |
| 22590 | Arthrodesis, posterior technique, craniocervical (occiput-C2) |
| 22595 | Arthrodesis, posterior technique, atlas-axis (C1-C2) |
| 22600 | Arthrodesis, posterior or posterolateral technique, single level; cervical below C2 segment |
| 22610 | Arthrodesis, posterior or posterolateral technique, single level; thoracic (with lateral transverse technique, when performed) |
| 22612 | Arthrodesis, posterior or posterolateral technique, single level; lumbar (with lateral transverse technique, when performed) |
| 22630 | Arthrodesis, posterior interbody technique, including laminectomy and/or discectomy to prepare interspace (other than for decompression), single interspace; lumbar |
| 22633 | Arthrodesis, combined posterior or posterolateral technique with posterior interbody technique including laminectomy and/or discectomy sufficient to prepare interspace (other than for decompression), single interspace and segment; lumbar |
| 22800 | Arthrodesis, posterior, for spinal deformity, with or without cast; up to 6 vertebral segments |
| 22802 | Arthrodesis, posterior, for spinal deformity, with or without cast; 7 to 12 vertebral segments |
| 22804 | Arthrodesis, posterior, for spinal deformity, with or without cast; 13 or more vertebral segments |
| 22808 | Arthrodesis, anterior, for spinal deformity, with or without cast; 2 to 3 vertebral segments |
| 22810 | Arthrodesis, anterior, for spinal deformity, with or without cast; 4 to 7 vertebral segments |
| 22812 | Arthrodesis, anterior, for spinal deformity, with or without cast; 8 or more vertebral segments |
| 27280 | Arthrodesis, open, sacroiliac joint, including obtaining bone graft, including instrumentation, when performed |
| 23615 | Open treatment of proximal humeral (surgical or anatomical neck) fracture, includes internal fixation, when performed, includes repair of tuberosity(s), when performed; |
| 23616 | Open treatment of proximal humeral (surgical or anatomical neck) fracture, includes internal fixation, when performed, includes repair of tuberosity(s), when performed; with proximal humeral prosthetic replacement |
| 23630 | Open treatment of greater humeral tuberosity fracture, includes internal fixation, when performed |
| 23670 | Open treatment of shoulder dislocation, with fracture of greater humeral tuberosity, includes internal fixation, when performed |
| 23680 | Open treatment of shoulder dislocation, with surgical or anatomical neck fracture, includes internal fixation, when performed |
| 24515 | Open treatment of humeral shaft fracture with plate/screws, with or without cerclage |
| 24516 | Treatment of humeral shaft fracture, with insertion of intramedullary implant, with or without cerclage and/or locking screws |
| 24545 | Open treatment of humeral supracondylar or transcondylar fracture, includes internal fixation, when performed; without intercondylar extension |
| 24546 | Open treatment of humeral supracondylar or transcondylar fracture, includes internal fixation, when performed; with intercondylar extension |
| 24575 | Open treatment of humeral epicondylar fracture, medial or lateral, includes internal fixation, when performed |
| 24579 | Open treatment of humeral condylar fracture, medial or lateral, includes internal fixation, when performed |
| 24586 | Open treatment of periarticular fracture and/or dislocation of the elbow (fracture distal humerus and proximal ulna and/or proximal radius); |
| 24587 | Open treatment of periarticular fracture and/or dislocation of the elbow (fracture distal humerus and proximal ulna and/or proximal radius); with implant arthroplasty |
| 24635 | Open treatment of Monteggia type of fracture dislocation at elbow (fracture proximal end of ulna with dislocation of radial head), includes internal fixation, when performed |
| 24665 | Open treatment of radial head or neck fracture, includes internal fixation or radial head excision, when performed; |
| 24666 | Open treatment of radial head or neck fracture, includes internal fixation or radial head excision, when performed; with radial head prosthetic replacement |
| 24685 | Open treatment of ulnar fracture, proximal end (eg, olecranon or coronoid process[es]), includes internal fixation, when performed |
| 25337 | Reconstruction for stabilization of unstable distal ulna or distal radioulnar joint, secondary by soft tissue stabilization (eg, tendon transfer, tendon graft or weave, or tenodesis) with or without open reduction of distal radioulnar joint |
| 25515 | Open treatment of radial shaft fracture, includes internal fixation, when performed |
| 25525 | Open treatment of radial shaft fracture, includes internal fixation, when performed, and open treatment of distal radioulnar joint dislocation (Galeazzi fracture/ dislocation), includes percutaneous skeletal fixation when performed |
| 25526 | Open treatment of radial shaft fracture, includes internal fixation, when performed, and open treatment of distal radioulnar joint dislocation (Galeazzi fracture/ dislocation), includes internal fixation, when performed, includes repair of triangular fibrocartilage complex |
| 25545 | Open treatment of ulnar shaft fracture, includes internal fixation, when performed |
| 25574 | Open treatment of radial AND ulnar shaft fractures, with internal fixation, when performed; of radius OR ulna |
| 25575 | Open treatment of radial AND ulnar shaft fractures, with internal fixation, when performed; of radius AND ulna |
| 25607 | Open treatment of distal radial extra-articular fracture or epiphyseal separation, with internal fixation |
| 25608 | Open treatment of distal radial intra-articular fracture or epiphyseal separation; with internal fixation of 2 fragments |
| 25609 | Open treatment of distal radial intra-articular fracture or epiphyseal separation; with internal fixation of 3 or more fragments |
| 25652 | Open treatment of ulnar styloid fracture |
| 27177 | Open treatment of slipped femoral epiphysis; single or multiple pinning or bone graft (includes obtaining graft) |
| 27178 | Open treatment of slipped femoral epiphysis; closed manipulation with single or multiple pinning |
| 27179 | Open treatment of slipped femoral epiphysis; osteoplasty of femoral neck (Heyman type procedure) |
| 27181 | Open treatment of slipped femoral epiphysis; osteotomy and internal fixation |
| 27244 | Treatment of intertrochanteric, peritrochanteric, or subtrochanteric femoral fracture; with plate/screw type implant, with or without cerclage |
| 27245 | Treatment of intertrochanteric, peritrochanteric, or subtrochanteric femoral fracture; with intramedullary implant, with or without interlocking screws and/or cerclage |
| 27248 | Open treatment of greater trochanteric fracture, includes internal fixation, when performed |
| 27254 | Open treatment of hip dislocation, traumatic, with acetabular wall and femoral head fracture, with or without internal or external fixation |
| 27269 | Open treatment of femoral fracture, proximal end, head, includes internal fixation, when performed |
| 27506 | Open treatment of femoral shaft fracture, with or without external fixation, with insertion of intramedullary implant, with or without cerclage and/or locking screws |
| 27507 | Open treatment of femoral shaft fracture with plate/screws, with or without cerclage |
| 27511 | Open treatment of femoral supracondylar or transcondylar fracture without intercondylar extension, includes internal fixation, when performed |
| 27513 | Open treatment of femoral supracondylar or transcondylar fracture with intercondylar extension, includes internal fixation, when performed |
| 27514 | Open treatment of femoral fracture, distal end, medial or lateral condyle, includes internal fixation, when performed |
| 27519 | Open treatment of distal femoral epiphyseal separation, includes internal fixation, when performed |
| 27535 | Open treatment of tibial fracture, proximal (plateau); unicondylar, includes internal fixation, when performed |
| 27536 | Open treatment of tibial fracture, proximal (plateau); bicondylar, with or without internal fixation |
| 27540 | Open treatment of intercondylar spine(s) and/or tuberosity fracture(s) of the knee, includes internal fixation, when performed |
| 27758 | Open treatment of tibial shaft fracture (with or without fibular fracture), with plate/screws, with or without cerclage |
| 27759 | Treatment of tibial shaft fracture (with or without fibular fracture) by intramedullary implant, with or without interlocking screws and/or cerclage |
| 27766 | Open treatment of medial malleolus fracture, includes internal fixation, when performed |
| 27769 | Open treatment of posterior malleolus fracture, includes internal fixation, when performed |
| 27784 | Open treatment of proximal fibula or shaft fracture, includes internal fixation, when performed |
| 27792 | Open treatment of distal fibular fracture (lateral malleolus), includes internal fixation, when performed |
| 27814 | Open treatment of bimalleolar ankle fracture (eg, lateral and medial malleoli, or lateral and posterior malleoli, or medial and posterior malleoli), includes internal fixation, when performed |
| 27822 | Open treatment of trimalleolar ankle fracture, includes internal fixation, when performed, medial and/or lateral malleolus; without fixation of posterior lip |
| 27823 | Open treatment of trimalleolar ankle fracture, includes internal fixation, when performed, medial and/or lateral malleolus; with fixation of posterior lip |
| 27826 | Open treatment of fracture of weight bearing articular surface/portion of distal tibia (eg, pilon or tibial plafond), with internal fixation, when performed; of fibula only |
| 27827 | Open treatment of fracture of weight bearing articular surface/portion of distal tibia (eg, pilon or tibial plafond), with internal fixation, when performed; of tibia only |
| 27828 | Open treatment of fracture of weight bearing articular surface/portion of distal tibia (eg, pilon or tibial plafond), with internal fixation, when performed; of both tibia and fibula |
| 27829 | Open treatment of distal tibiofibular joint (syndesmosis) disruption, includes internal fixation, when performed |
| 27438 | Arthroplasty, patella; with prosthesis |
| 27440 | Arthroplasty, knee, tibial plateau |
| 27441 | Arthroplasty, knee, tibial plateau; with debridement and partial synovectomy |
| 27442 | Arthroplasty, femoral condyles or tibial plateau(s), knee |
| 27443 | Arthroplasty, femoral condyles or tibial plateau(s), knee; with debridement and partial synovectomy |
| 27445 | Arthroplasty, knee, hinge prosthesis (eg, Walldius type) |
| 27446 | Arthroplasty, knee, condyle and plateau; medial OR lateral compartment |
| 27447 | Arthroplasty, knee, condyle and plateau; medial AND lateral compartments with or without patella resurfacing (total knee arthroplasty) |
| 27486 | Revision of total knee athroplasty, with or without allograft, one component |
| 27487 | Revision of total knee arthroplasty, with or without allograft; femoral and entire tibial component |
| 22220 | Osteotomy of spine, including discectomy, anterior approach, single vertebral segment; cervical |
| 22222 | Osteotomy of spine, including discectomy, anterior approach, single vertebral segment; thoracic |
| 22224 | Osteotomy of spine, including discectomy, anterior approach, single vertebral segment; lumbar |
| 22856 | Total disc arthroplasty (artificial disc), anterior approach, including discectomy with end plate preparation (includes osteophytectomy for nerve root or spinal cord decompression and microdissection); single interspace, cervical |
| 22857 | Total disc arthroplasty (artificial disc), anterior approach, including discectomy to prepare interspace (other than for decompression), single interspace, lumbar |
| 22861 | Revision including replacement of total disc arthroplasty (artificial disc), anterior approach, single interspace; cervical |
| 22862 | Revision including replacement of total disc arthroplasty (artificial disc), anterior approach, single interspace; lumbar |
| 22867 | Insertion of interlaminar/interspinous process stabilization/distraction device, without fusion, including image guidance when performed, with open decompression, lumbar; single level |
| 62287 | Decompression procedure, percutaneous, of nucleus pulposus of intervertebral disc, any method utilizing needle based technique to remove disc material under fluoroscopic imaging or other form of indirect visualization, with the use of an endoscope, with discography and/or epidural injection(s) at the treated level(s), when performed, single or multiple levels, lumbar |
| 62351 | Implantation, revision or repositioning of tunneled intrathecal or epidural catheter, for long-term medication administration via an external pump or implantable reservoir/infusion pump; with laminectomy |
| 62380 | Endoscopic decompression of spinal cord, nerve root(s), including laminectomy, partial facetectomy, foraminotomy, discectomy and/or excision of herniated intervertebral disc, 1 interpace, lumbar |
| 63001 | Laminectomy with exploration and/or decompression of spinal cord and/or cauda equina, without facetectomy, foraminotomy or discectomy (eg, spinal stenosis), 1 or 2 vertebral segments; cervical |
| 63003 | Laminectomy with exploration and/or decompression of spinal cord and/or cauda equina, without facetectomy, foraminotomy or discectomy (eg, spinal stenosis), 1 or 2 vertebral segments; thoracic |
| 63005 | Laminectomy with exploration and/or decompression of spinal cord and/or cauda equina, without facetectomy, foraminotomy or discectomy (eg, spinal stenosis), 1 or 2 vertebral segments; lumbar, except for spondylolisthesis |
| 63011 | Laminectomy with exploration and/or decompression of spinal cord and/or cauda equina, without facetectomy, foraminotomy or discectomy (eg, spinal stenosis), 1 or 2 vertebral segments; sacral |
| 63012 | Laminectomy with removal of abnormal facets and/or pars inter-articularis with decompression of cauda equina and nerve roots for spondylolisthesis, lumbar (Gill type procedure) |
| 63015 | Laminectomy with exploration and/or decompression of spinal cord and/or cauda equina, without facetectomy, foraminotomy or discectomy (eg, spinal stenosis), more than 2 vertebral segments; cervical |
| 63016 | Laminectomy with exploration and/or decompression of spinal cord and/or cauda equina, without facetectomy, foraminotomy or discectomy (eg, spinal stenosis), more than 2 vertebral segments; thoracic |
| 63017 | Laminectomy with exploration and/or decompression of spinal cord and/or cauda equina, without facetectomy, foraminotomy or discectomy (eg, spinal stenosis), more than 2 vertebral segments; lumbar |
| 63020 | Laminotomy (hemilaminectomy), with decompression of nerve root(s), including partial facetectomy, foraminotomy and/or excision of herniated intervertebral disc; 1 interspace, cervical |
| 63030 | Laminotomy (hemilaminectomy), with decompression of nerve root(s), including partial facetectomy, foraminotomy and/or excision of herniated intervertebral disc; 1 interspace, lumbar |
| 63035 | Laminotomy (hemilaminectomy), with decompression of nerve root(s), including partial facetectomy, foraminotomy and/or excision of herniated intervertebral disc; each No changeitional interspace, cervical or lumbar (List separately in No changeition to code for primary procedure) |
| 63040 | Laminotomy (hemilaminectomy), with decompression of nerve root(s), including partial facetectomy, foraminotomy and/or excision of herniated intervertebral disc, reexploration, single interspace; cervical |
| 63042 | Laminotomy (hemilaminectomy), with decompression of nerve root(s), including partial facetectomy, foraminotomy and/or excision of herniated intervertebral disc, reexploration, single interspace; lumbar |
| 63045 | Laminectomy, facetectomy and foraminotomy (unilateral or bilateral with decompression of spinal cord, cauda equina and/or nerve root[s], [eg, spinal or lateral recess stenosis]), single vertebral segment; cervical |
| 63046 | Laminectomy, facetectomy and foraminotomy (unilateral or bilateral with decompression of spinal cord, cauda equina and/or nerve root[s], [eg, spinal or lateral recess stenosis]), single vertebral segment; thoracic |
| 63047 | Laminectomy, facetectomy and foraminotomy (unilateral or bilateral with decompression of spinal cord, cauda equina and/or nerve root[s], [eg, spinal or lateral recess stenosis]), single vertebral segment; lumbar |
| 63048 | Laminectomy, facetectomy and foraminotomy (unilateral or bilateral with decompression of spinal cord, cauda equina and/or nerve root[s], [eg, spinal or lateral recess stenosis]), single vertebral segment; each No changeitional segment, cervical, thoracic, or lumbar (List separately in No changeition to code for primary procedure) |
| 63050 | Laminoplasty, cervical, with decompression of the spinal cord, 2 or more vertebral segments; |
| 63051 | Laminoplasty, cervical, with decompression of the spinal cord, 2 or more vertebral segments; with reconstruction of the posterior bony elements (including the application of bridging bone graft and non-segmental fixation devices [eg, wire, suture, mini-plates], when performed) |
| 63055 | Transpedicular approach with decompression of spinal cord, equina and/or nerve root(s) (eg, herniated intervertebral disc), single segment; thoracic |
| 63056 | lumbar (including transfacet, or lateral extraforaminal approach) (eg, far lateral herniated intervertebral disc |
| 63064 | Costovertebral approach with decompression of spinal cord or nerve root(s), (eg, herniated intervertebral disc), thoracic; single segment |
| 63075 | Discectomy, anterior, with decompression of spinal cord and/or nerve root(s), including osteophytectomy; cervical, single interspace |
| 63077 | Discectomy, anterior, with decompression of spinal cord and/or nerve root(s), including osteophytectomy; thoracic, single interspace |
| 63081 | Vertebral corpectomy (vertebral body resection), partial or complete, anterior approach with decompression of spinal cord and/or nerve root(s); cervical, single segment |
| 63082 | Vertebral corpectomy (vertebral body resection), partial or complete, anterior approach with decompression of spinal cord and/or nerve root(s); cervical, each No changeitional segment (List separately in No changeition to code for primary procedure) |
| 63085 | Vertebral corpectomy (vertebral body resection), partial or complete, transthoracic approach with decompression of spinal cord and/or nerve root(s); thoracic, single segment |
| 63086 | Vertebral corpectomy (vertebral body resection), partial or complete, transthoracic approach with decompression of spinal cord and/or nerve root(s); thoracic, each No changeitional segment (List separately in No changeition to code for primary procedure) |
| 63087 | Vertebral corpectomy (vertebral body resection), partial or complete, combined thoracolumbar approach with decompression of spinal cord, cauda equina or nerve root(s), lower thoracic or lumbar; single segment |
| 63088 | Vertebral corpectomy (vertebral body resection), partial or complete, combined thoracolumbar approach with decompression of spinal cord, cauda equina or nerve root(s), lower thoracic or lumbar; each No changeitional segment (List separately in No changeition to code for primary procedure) |
| 63090 | Vertebral corpectomy (vertebral body resection), partial or complete, transperitoneal or retroperitoneal approach with decompression of spinal cord, cauda equina or nerve root(s), lower thoracic, lumbar, or sacral; single segment |
| 63091 | Vertebral corpectomy (vertebral body resection), partial or complete, transperitoneal or retroperitoneal approach with decompression of spinal cord, cauda equina or nerve root(s), lower thoracic, lumbar, or sacral; each No changeitional segment (List separately in No changeition to code for primary procedure) |
| 63101 | Vertebral corpectomy (vertebral body resection), partial or complete, lateral extracavitary approach with decompression of spinal cord and/or nerve root(s) (eg, for tumor or retropulsed bone fragments); thoracic, single segment |
| 63102 | Vertebral corpectomy (vertebral body resection), partial or complete, lateral extracavitary approach with decompression of spinal cord and/or nerve root(s) (eg, for tumor or retropulsed bone fragments); lumbar, single segment |
| 63103 | Vertebral corpectomy (vertebral body resection), partial or complete, lateral extracavitary approach with decompression of spinal cord and/or nerve root(s) (eg, for tumor or retropulsed bone fragments); thoracic or lumbar, each No changeitional segment (List separately in No changeition to code for primary procedure) |
| 0202T | Posterior vertebral joint(s) arthroplasty (eg, facet joint[s] replacement), including facetectomy, laminectomy, foraminotomy, and vertebral column fixation, injection of bone cement, when performed, including fluoroscopy, single level, lumbar spine |
| 0219T | Placement of a posterior intrafacet implant(s), unilateral or bilateral, including imaging and placement of bone graft(s) or synthetic device(s), single level; cervical |
| 0220T | Placement of a posterior intrafacet implant(s), unilateral or bilateral, including imaging and placement of bone graft(s) or synthetic device(s), single level; thoracic |
| 0221T | Placement of a posterior intrafacet implant(s), unilateral or bilateral, including imaging and placement of bone graft(s) or synthetic device(s), single level; lumbar |

CPT, Current procedural terminology.
